# Percutaneous Paravalvular Leak Closure of the EVOQUE Transcatheter Tricuspid Valve Replacement System

**DOI:** 10.1016/j.shj.2025.100421

**Published:** 2025-01-27

**Authors:** Gennaro Giustino, James C. Lee, Brian P. O’Neill, Jonathan X. Fang, Bryan Zweig, Ahmad Jabri, Tiberio M. Frisoli, Pedro Engel, William W. O’Neill, Pedro A. Villablanca

**Affiliations:** Center for Structural Heart Disease, Henry Ford Health System, Detroit, Michigan, USA

**Keywords:** EVOQUE, PVL, Tricuspid regurgitation

## Abstract

• Transcatheter closure of paravalvular leak of the EVOQUE valve is feasible and can be done from a transfemoral or transjugular approach.
• Preoperative cardiac computed tomography is useful to characterize the location and size of the paravalvular leak defects.
• The mechanisms of late paravalvular leak are unclear, but the progressive right ventricular dilatation and ellipsoid deformation of the tricuspid annulus may contribute to the development of leaks in the septal and lateral positions.

A 57-year-old man with severe tricuspid regurgitation, surgical mechanical aortic valve replacement, and atrial fibrillation underwent successful transcatheter tricuspid valve replacement (TTVR) with the 56 mm EVOQUE system. Transthoracic echocardiogram post-TTVR demonstrated trivial paravalvular leak (PVL) and a transvalvular gradient of 3.6 mmHg (Online Video 1). He presented 1 year later with worsening fatigue, dyspnea on exertion, and peripheral edemas. Repeat transesophageal echocardiography and cardiac computed tomography demonstrated the presence of two new paravalvular leaks (PVLs), one septal-anterior at 2 o’clock of at least moderate severity (Figure 1a-c) and one postero-lateral moderate-to-severe at 8 o’clock (Figure 1d-f), with unchanged transvalvular gradients.

**Figure 1:**
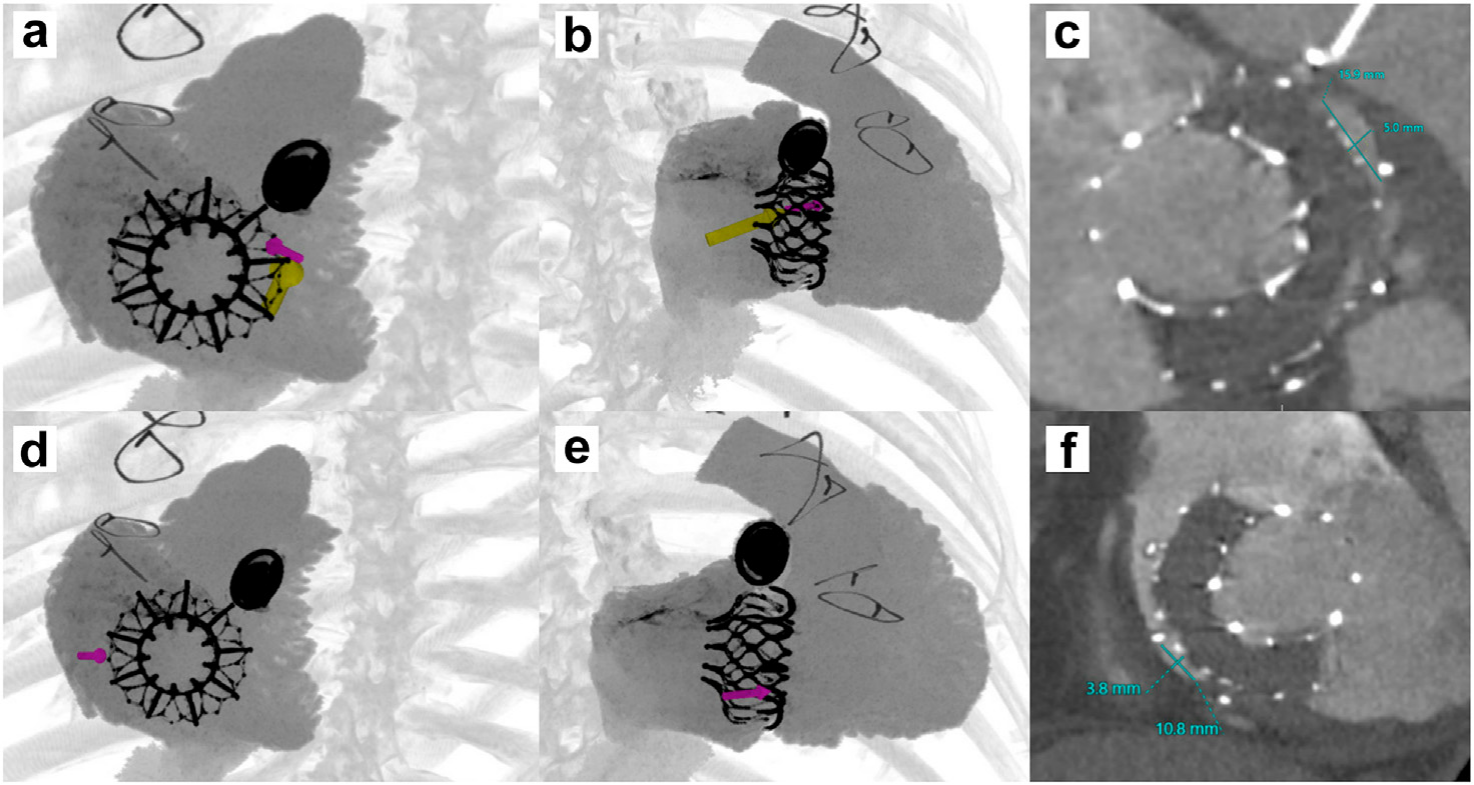
Preoperative cardiac computed tomography. (a-c) Antero-septal PVL at 2 o’clock and (d-f) postero-lateral PVL at 8 o’clock. Abbreviation: PVL, paravalvular leak.

After multidisciplinary team meeting, we opted for transcatheter closure of the PVLs. The procedure was performed under general anesthesia with transesophageal echocardiography and 4D-intracardiac echocardiographic guidance. Right internal jugular access was obtained with a 26-Fr DrySeal sheath. The septal-anterior defect (Online Video 2) was crossed with a straight-tip glidewire within a medium-curved Agilis catheter, which was then snared and externalized from the left femoral vein (Figure 2a-c). Using the veno-venous rail, an 18-mm ventricular septal occluder device was deployed across the defect, keeping a 0.035 Confida wire across the defect as a safety wire (Figure 2d-e). The postero-lateral defect (Online Video 3) was crossed in a similar fashion, but the glidewire was advanced in the pulmonary artery and then exchanged with a Lunderquist wire (Figure 2f-g). Another Lunderquist and an Amplatz super-stiff wire were sent across the defect into the pulmonary artery, and three 18-mm ventricular septal occluder devices were deployed across the defect (Figure 2h-m). Final intracardiac echocardiographic images are illustrated in Online Video 4 and Figure 3. The next-day transthoracic echocardiogram demonstrated no PVL and a transvalvular gradient of 4.4 mmHg (Online Video 5). Representative images of post-PVL-closure cardiac computed tomography are illustrated in Figure 4a-c. The hospital course was uneventful, and the patient was discharged home on postprocedure day 1.

**Figure 2:**
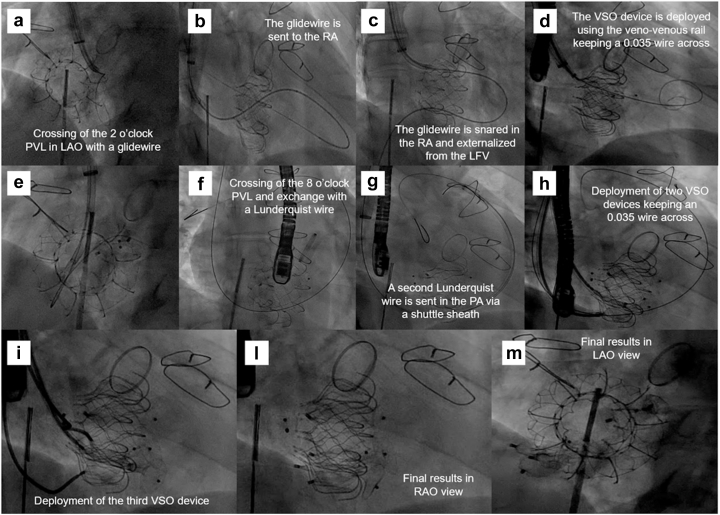
Procedural steps for PVL closure. (a-e) Crossing and closure of the antero-septal PVL and (f-i) the postero-lateral PVL. (l and m) Final results. Abbreviations: LAO, left antior oblique; LFV, left femoral vein; PA, pulmonary artery; PVL, paravalvular leak; RA, right atrium; RAO, right anterior oblique; VSO, ventricular septal occluder.

**Figure 3:**
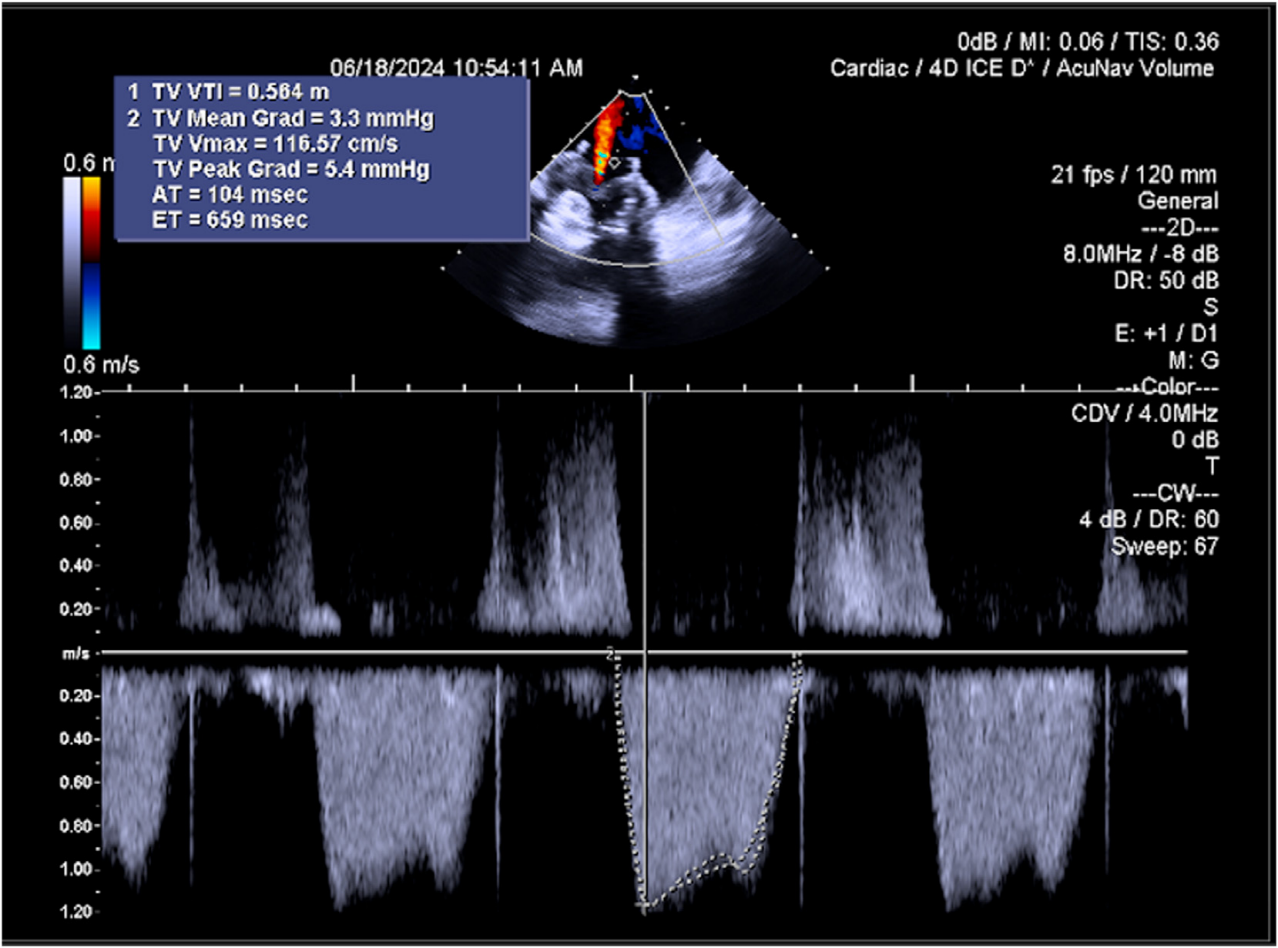
Final intracardiac echocardiographic imaging after PVL closure. Abbreviations: AT, acceleration time; CDV, Color Doppler Velocity; CW, continuous wave; ET, ejection time; PVL, paravalvular leak; TV, tricuspid valve; Vmax, peak aortic valve velocity; VTI, velocity time interval; 2D, two-dimensional.

**Figure 4:**
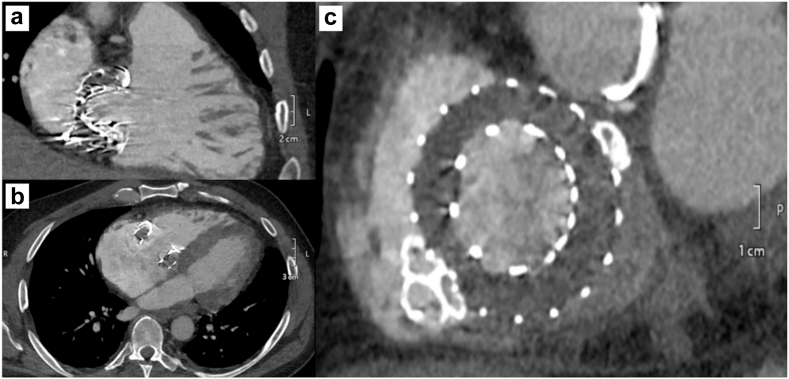
Postoperative cardiac computed tomography. (a-c) Displays the relation between the VSO devices and the EVOQUE valve. Abbreviation: VSO, ventricular septal occluder.

The current report is the first description of the preoperative work-up and percutaneous technique to treat post-EVOQUE PVLs. The mechanisms of late PVLs post-EVOQUE currently remain unclear; however, their incidence may increase over time with the adoption of TTVR. As with progressive maladaptive remodeling of the right ventricle and subsequent ellipsoid annular enlargement, we speculate that late PVLs post-TTVR may more frequently occur in the septal and lateral positions. In fact, in our report, PVLs were located at the 2 and 8 o’clock positions, which were not observed after the index EVOQUE implantation. We opted to create a veno-venous rail to cross the antero-septal defect but not the posterior-lateral as we needed greater support to send the delivery sheath across this defect due to a more unfavorable angulation coming from the right internal jugular vein. Conversely, the delivery sheath was easily sent across the posterior-lateral defect by having a stiff wire in the pulmonary artery due to a more favorable trajectory from the right internal jugular vein.

## Consent Statement

Procedural consent was obtained from the patient as per local standard of care. No investigational or research consent was obtained as this is not an investigational procedure.

## Funding

The authors have no funding to report.

## Disclosure Statement

Dr Giustino is a consultant and proctor for Edwards Lifesciences. Dr Frisoli is a clinical proctor for Edwards Lifesciences, Abbott, Boston Scientific, and Medtronic and reports a relationship with Henry Ford Hospital that includes consulting or advisory. Dr Brian O'Neill is a consultant to Abbott, Edwards Lifesciences, and Medtronic; receives research support from 10.13039/100006520Edwards Lifesciences assigned to employer Henry Ford Health; and reports a relationship with Henry Ford Hospital that includes consulting or advisory. Dr William O'Neill is a consultant to Abiomed, Medtronic, and Boston Scientific and reports a relationship with Henry Ford Hospital that includes consulting or advisory. Dr Villablanca is a consultant for Edwards Lifesciences and Teleflex and reports a relationship with Henry Ford Hospital that includes consulting or advisory. The other authors had no conflicts to declare.

## Supplementary Material

Online Video 1 Transthoracic echocardiogram after the index EVOQUE implantation showing no significant gradient and trace PVL.

Online Video 2 Demonstration and crossing of the antero-septal PVL with 3D-ICE.

Online Video 3 Demonstration of the postero-lateral PVL with 3D-ICE.

Online Video 4 Final 3D-ICE after PVL closure.

Online Video 5 Next-day transthoracic echocardiogram demonstrating no PVL.

